# High-Grade Temporal Bone Sarcoma Mimicking Cholesteatoma During Pregnancy: A Diagnostic Pitfall and Multidisciplinary Challenge

**DOI:** 10.7759/cureus.108462

**Published:** 2026-05-07

**Authors:** Hillary Lizarraga, José Gonzalo Bravo Quiroz, José Manuel García Romero, Dina Fabiola Gonzalez Sanchez, Diego D Linares Pinetta

**Affiliations:** 1 Otolaryngology-Head and Neck Surgery, Hospital General Dr. Manuel Gea Gonzalez, Mexico City, MEX; 2 General Practice, Universidad Autónoma de Querétaro, Querétaro, MEX; 3 Plastic Surgery, University Hospital Coventry & Warwickshire, Coventry, GBR; 4 Otolaryngology-Head and Neck Surgery, Hospital General Dr. Manuel Gea Gonzalez, Mexico city, MEX

**Keywords:** cholesteatome, craniofacial osteosarcoma, osteosarcoma (os), pregnant patient, spindle cell and epithelioid sarcoma

## Abstract

Osteosarcoma of the head and neck is an uncommon malignancy with heterogeneous clinical and radiological presentations, often leading to diagnostic challenges. Its occurrence during pregnancy further complicates management due to the need to balance maternal and fetal outcomes.

We report the case of a 23-year-old pregnant woman who initially presented with right-sided otalgia, managed as otitis externa. She subsequently developed a rapidly progressive, highly vascular mass protruding through the external auditory canal, associated with conductive hearing loss and vestibular symptoms. Magnetic resonance imaging revealed an aggressive lesion involving the external auditory canal, middle ear, and mastoid air cells and extending to the dura of the middle and posterior cranial fossae.

An initial biopsy performed at 17.5 weeks of gestation was suggestive of cholesteatoma. However, due to clinical progression, surgical resection via mastoidectomy was performed. Definitive histopathological analysis revealed a high-grade spindle cell and epithelioid sarcoma with osteoid formation, consistent with osteosarcoma.

The patient developed early recurrence with intracranial progression, requiring multidisciplinary management. Radiotherapy was initiated at 28 weeks of gestation, followed by termination of pregnancy at 29 weeks due to maternal oncologic progression and oligohydramnios. Systemic chemotherapy was subsequently administered, achieving a partial response.

In our case, the lesion was initially approached as a benign otologic condition, which delayed definitive diagnosis despite imaging findings suggestive of aggressive disease. Both clinical evaluation and initial histopathological assessment failed to establish the correct diagnosis. This case also underscores the complexity of oncologic decision-making during pregnancy and the critical importance of a multidisciplinary approach.

## Introduction

Osteosarcoma is a malignant mesenchymal tumor defined by the production of osteoid by neoplastic cells, representing one of the most common primary bone malignancies [1]. Its biological behavior is heterogeneous, often characterized by aggressive growth, high-grade histology, and a propensity for local invasion and recurrence [2].

Despite its relative frequency in long bones, involvement of the head and neck region is rare, accounting for less than 10% of all cases [3]. In this anatomical location, osteosarcomas present unique diagnostic and therapeutic challenges due to complex regional anatomy and the difficulty in achieving adequate surgical margins [3,4].

Clinically, head and neck osteosarcomas may present with nonspecific symptoms that overlap with more common benign or inflammatory conditions, contributing to delays in diagnosis [4]. Radiological evaluation plays a key role in the assessment of these lesions; however, imaging findings are variable and may lack specificity, frequently mimicking other pathologies [5].

This diagnostic challenge is even more pronounced in lesions involving the temporal bone, which are exceedingly rare and may resemble more common otologic entities such as cholesteatoma, particularly when presenting with conductive hearing loss and external auditory canal involvement [6]. Furthermore, osteosarcoma and related sarcomas may exhibit significant clinical and histological heterogeneity, increasing the risk of misinterpretation, especially when biopsy samples are limited or non-representative [7].

The coexistence of malignancy and pregnancy introduces an additional layer of complexity in clinical decision-making, as treatment strategies must balance maternal prognosis with fetal safety, often requiring individualized and multidisciplinary approaches.

We present a case of high-grade temporal bone sarcoma initially misdiagnosed as cholesteatoma in a pregnant patient, highlighting the diagnostic pitfalls, aggressive clinical behavior, and therapeutic challenges inherent to this rare entity.

## Case presentation

**Figure 1:**
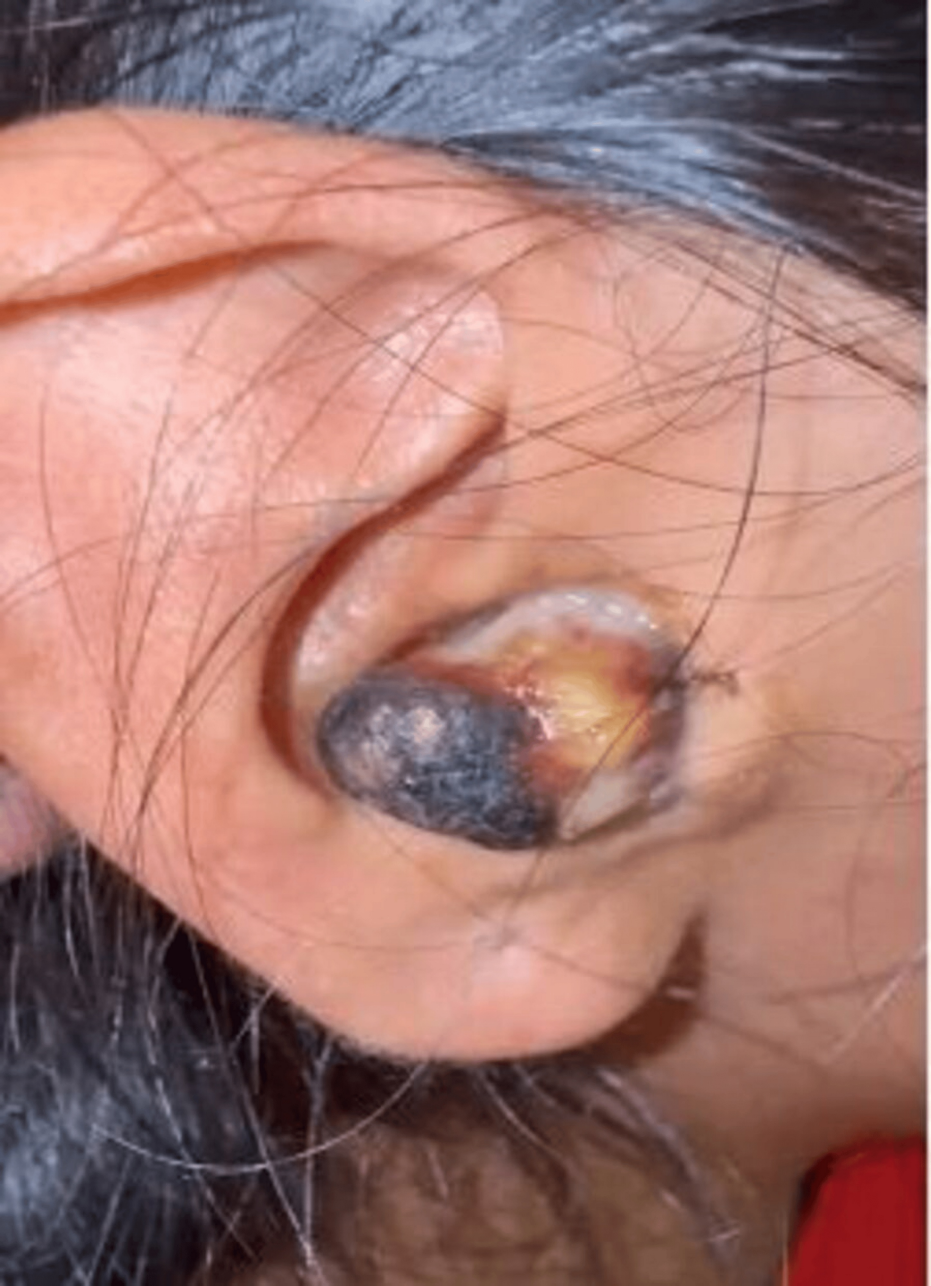
Brownish-whitish mass with central necrotic areas, protruding through the right external auditory canal

A 23-year-old previously healthy woman presented with right-sided otalgia, initially managed as otitis externa. Over time, she developed a progressively enlarging mass protruding through the external auditory canal. The lesion appeared highly vascularized, with a brownish-whitish coloration and areas of central necrosis, and bled easily upon manipulation (Figure 1).

These findings were associated with ipsilateral conductive hearing loss, headache, and vestibular symptoms, including vertigo. At the time of initial otolaryngology evaluation, the patient was 13 weeks pregnant.

**Figure 2:**
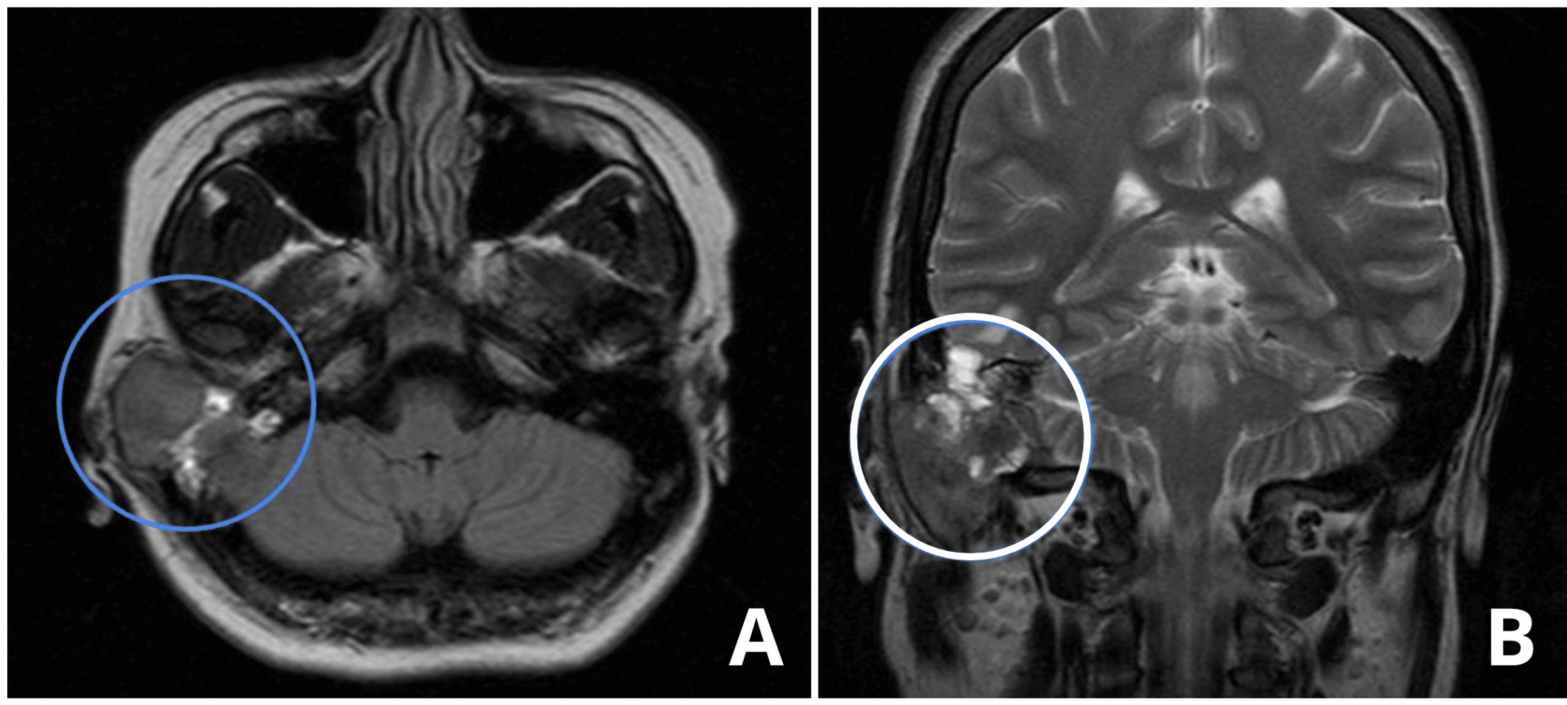
Brain MRI (non-contrast) A: Axial T1-weighted showing an expansile lesion with mixed signal intensity, predominantly of soft tissue (blue circle). The lesion involves the bony portion of the external auditory canal, affecting the anterior, posterior, and inferior walls, with an expansile and destructive pattern, extending to the temporal bone and middle ear. B: Coronal T2-weighted showing a lesion with a central component with signal intensity similar to soft tissue (white circle). The lesion extends intracranially, displacing the cerebellar hemisphere.

Magnetic resonance imaging demonstrated an expansile lesion involving the external auditory canal, middle ear, and mastoid air cells, with extension toward the dura of both the middle and posterior cranial fossae, without a clear plane of separation on MRI sequences. Dural involvement was therefore radiologically suspected; histological confirmation of dural invasion was not obtained, as the surgical approach did not include intradural exploration. These findings raised concern for an aggressive process (Figure 2).

Given the extent of the lesion and its apparent dural involvement, the initial differential diagnosis included both aggressive benign pathology, such as extensive cholesteatoma, and malignant neoplasms. However, the patient’s age and clinical presentation initially favored a benign etiology.

A biopsy was performed under sedation with continuous fetal monitoring at 17.5 weeks of gestation. Histopathological analysis of the limited sample was initially reported as compatible with cholesteatoma. However, the limited nature of the sample and the heterogeneous architecture of the lesion raised concern for a potential sampling error, particularly in the context of ongoing clinical deterioration.

Following the procedure, the patient experienced clinical deterioration, characterized by intense right-sided facial pain, progressive preauricular swelling resulting in facial asymmetry, and worsening hearing loss. In light of the clinical progression and discordance with the initial diagnosis, surgical management was pursued.

**Figure 3:**
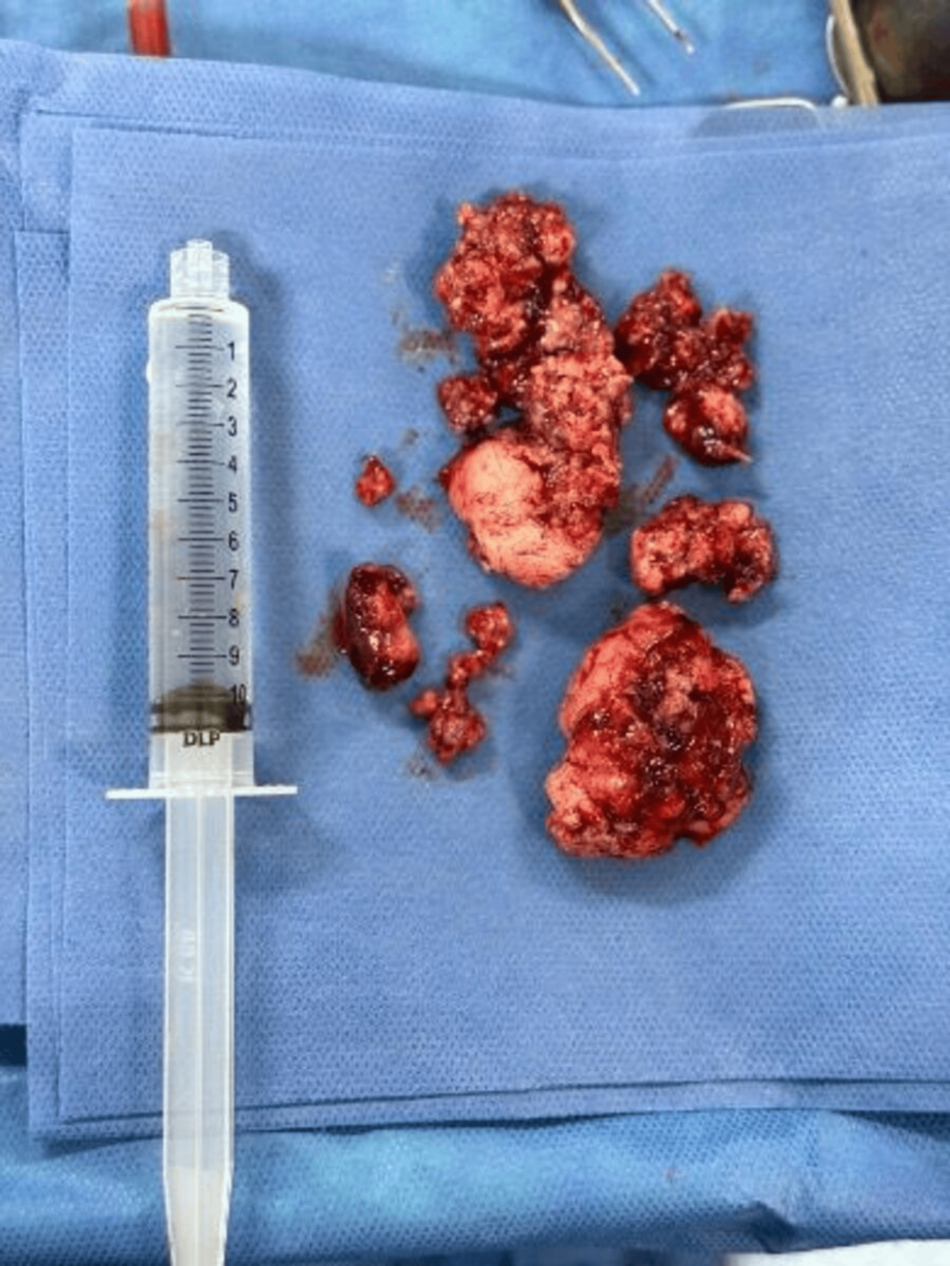
Surgical specimen Multiple irregular nodular tissue fragments. The external surface is irregular, lobulated, and opaque, with a brown-gray coloration and hemorrhagic areas and a soft consistency. The estimated total volume is approximately 120 cm³. The largest fragment measures 4.4 × 3.5 × 2 cm, and the smallest measures 0.4 × 0.3 × 0.2 cm.

A mastoidectomy was performed, achieving gross macroscopic resection of the lesion without intraoperative complications (Figure 3).

**Figure 4:**
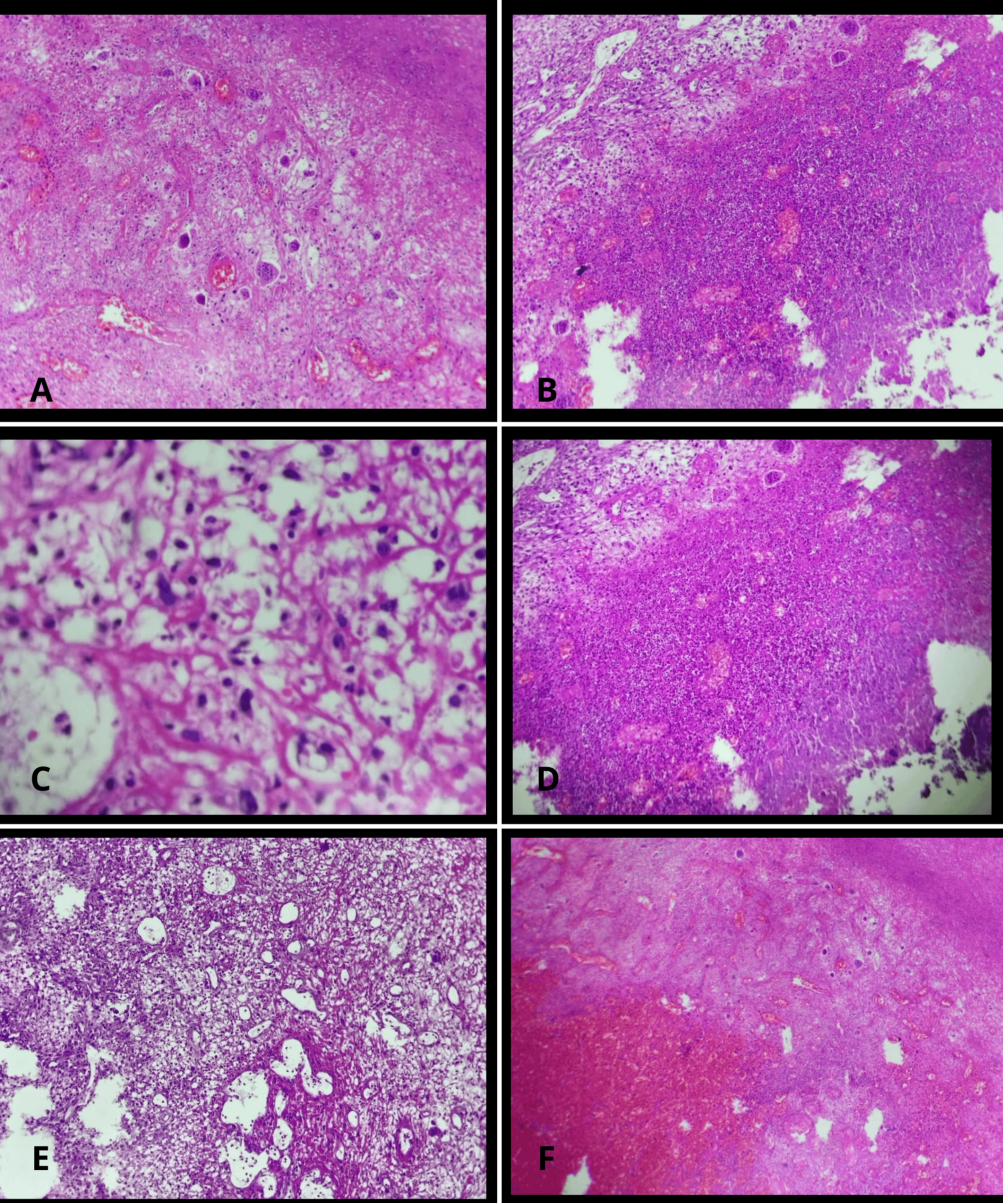
Histopathological findings A: Low-power photomicrograph (H&E stain) showing a highly cellular lesion composed of spindle-shaped cells arranged in a disorganized pattern, with areas of extracellular eosinophilic matrix suggestive of osteoid formation. Foci of hemorrhage and necrosis are also identified. B: Low- to intermediate-power view demonstrating extensive tumor cellularity with areas of necrosis and hemorrhage. A mixed inflammatory infiltrate is present within the tumor microenvironment. C: High-power photomicrograph revealing atypical cells with pleomorphic nuclei embedded in an eosinophilic extracellular matrix, compatible with osteoid production. Increased mitotic activity is noted. D: High-power view showing marked cellular atypia with spindle and epithelioid morphology, nuclear pleomorphism, and hyperchromasia, consistent with a high-grade malignant neoplasm. E: Intermediate-power image demonstrating areas of vascular proliferation with hemorrhagic foci and tumor infiltration, supporting the aggressive biological behavior of the lesion. F: Low-power photomicrograph illustrating a heterogeneous tumor architecture with alternating hypercellular and hypocellular areas, associated with necrosis and stromal changes.

Surgical margins were reported as negative on definitive histopathological analysis. Definitive histopathological analysis, however, revealed a high-grade spindle cell and epithelioid sarcoma with myofibroblastic differentiation and osteoid matrix formation, with approximately 30% tumor necrosis and a high mitotic index (25 mitoses per 1 mm²). Immunohistochemical studies showed positivity for actin, SATB2, and osteonectin and negativity for S100, CDK4, MDM2, and SOX10, supporting the diagnosis of osteosarcoma (Figure 4).

**Figure 5:**
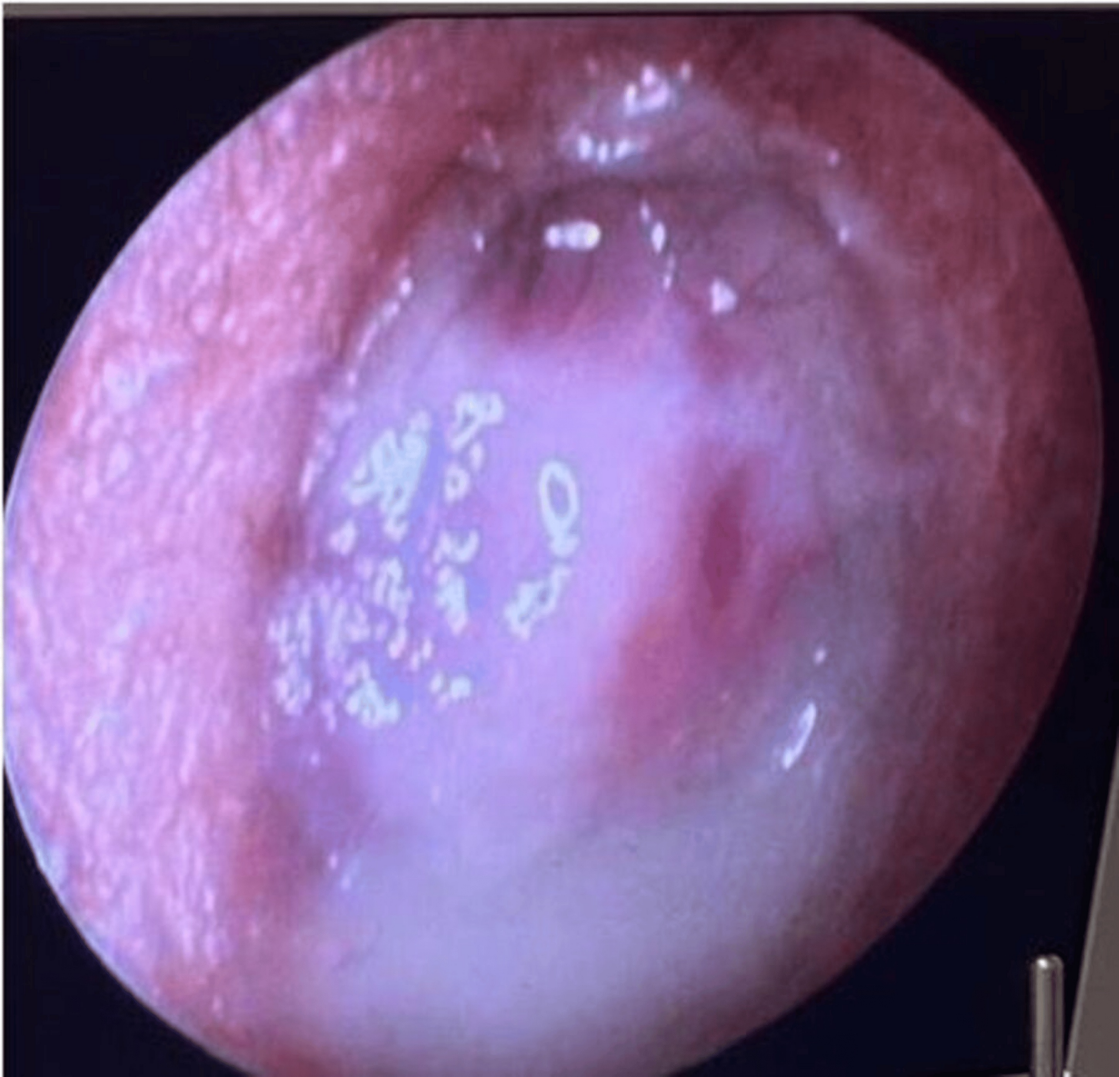
Rigid 0° endoscopy findings Occupying lesion of the right external auditory canal, with a pinkish-violet appearance, friable, highly vascularized, and associated with purulent otorrhea.

Two weeks after surgery, the patient developed early clinical recurrence, with rapid regrowth of the lesion within the external auditory canal and recurrence of vestibular symptoms (Figure 5). 

**Figure 6:**
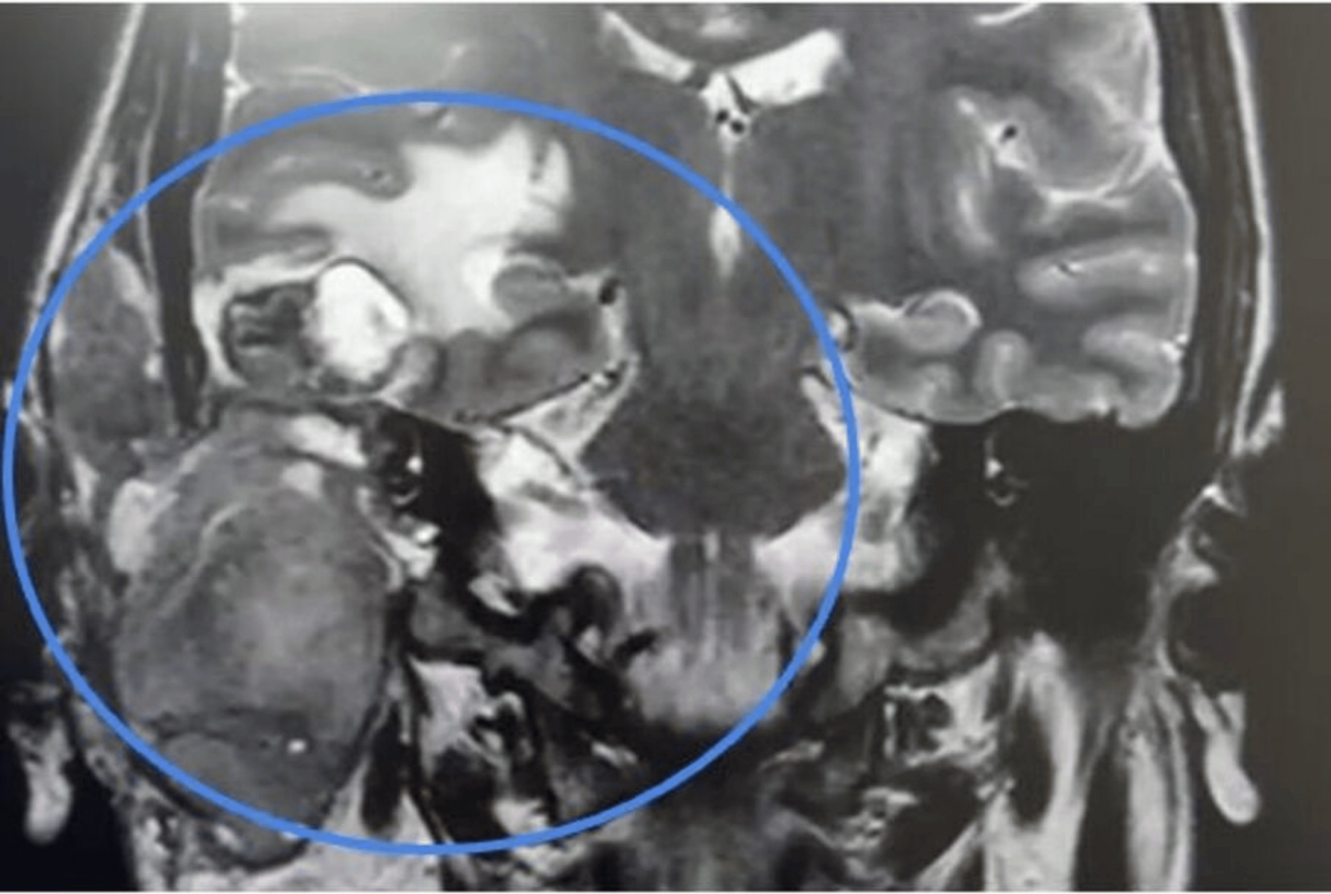
Follow-up brain MRI (non-contrast) Coronal T2-weighted images demonstrate an expansile and destructive lesion involving the temporal bone and middle ear (blue circle). A right temporal lobe lesion with mixed signal intensity and surrounding perilesional edema is also observed.

Follow-up MRI confirmed tumor progression with intracranial invasion (Figure 6).

Given the aggressive tumor behavior and gestational age, a multidisciplinary counseling process was conducted involving the patient, her family, the oncology team, obstetrics, and radiation oncology. The risks and potential benefits of radiotherapy during pregnancy were discussed in detail, and written informed consent was obtained prior to treatment initiation. Radiotherapy was initiated at 28 weeks of gestation. The patient received 37.5 Gy in 15 fractions. Physical fetal shielding was performed using lead blocks throughout the treatment course to minimize scatter radiation exposure to the uterus and fetus. Formal fetal dosimetric assessment was performed and confirmed fetal exposure within thresholds considered safe for fetal development, consistent with established radiobiological principles and below the 100 mGy threshold associated with deterministic fetal effects as referenced by the International Commission on Radiological Protection (ICRP). The precise dosimetric value is acknowledged as a limitation of this report.

Following partial response, an additional 33.75 Gy in 15 fractions was administered as a complementary dose. At 29 weeks of gestation, due to the development of oligohydramnios and ongoing maternal disease progression, the decision was made to proceed with termination of pregnancy via cesarean section. The neonate was born with a birth weight of 2,400 g and APGAR scores of 7/10. Neonatal intensive care unit admission was not required. No neonatal complications were identified, and the infant is currently under routine pediatric follow-up without reported complications to date.

Postpartum, systemic chemotherapy was initiated using a regimen based on doxorubicin and ifosfamide. At present, the patient shows significant clinical improvement, with tumor cytoreduction. She developed grade 1 radiodermatitis in the temporal region and radiotherapy-associated alopecia, both of which are being managed conservatively. She remains under follow-up pending further imaging evaluation.

## Discussion

Osteosarcoma of the head and neck is a rare and aggressive malignancy, with distinct clinical and biological behavior compared to its long bone counterpart [3]. These tumors are classically characterized by the production of osteoid by malignant mesenchymal cells and often exhibit high-grade histology and locally aggressive behavior [1,2].

Temporal bone involvement is exceptionally uncommon, with only isolated cases reported in the literature, which contributes to the difficulty in establishing early clinical suspicion [6]. In our case, the initial presentation closely resembled more common otologic conditions, including otitis externa and cholesteatoma. The presence of conductive hearing loss and a canal mass further reinforced this initial impression.

However, certain clinical features, such as the highly vascular and friable nature of the lesion, were atypical for cholesteatoma and, in retrospect, should have prompted earlier consideration of alternative diagnoses. The differential diagnosis of a highly vascularized external auditory canal mass in this clinical context should include glomus tumors (glomus jugulare or glomus tympanicum), squamous cell carcinoma, and high-grade sarcomas [5,6]. In the present case, a glomus tumor was considered less likely due to the absence of pulsatile tinnitus and the lack of the characteristic "salt-and-pepper" T1 signal pattern on MRI. Squamous cell carcinoma was considered unlikely given the patient's young age and the absence of established risk factors such as chronic otitis media or prior radiation exposure. These diagnoses were ultimately excluded by histopathological and immunohistochemical analysis.

Imaging findings in head and neck osteosarcoma are variable and often nonspecific, frequently overlapping with benign or inflammatory conditions, which may contribute to diagnostic delay [5]. Unlike many previously reported cases, which are often incidental or slowly progressive, our patient demonstrated rapid clinical deterioration following initial evaluation, emphasizing the aggressive nature of this tumor. The high mitotic index observed further supports this aggressive clinical behavior and explains the early recurrence. Notably, surgical margins had been reported as negative following mastoidectomy; the early recurrence observed within two weeks likely reflects the aggressive biological behavior of this high-grade sarcoma, with possible microscopic residual disease beyond the evaluated margins.

A key aspect of this case was the initial histopathological misinterpretation. Osteosarcoma is known for its marked histological heterogeneity, and limited biopsy samples may fail to demonstrate diagnostic features such as osteoid production, leading to potential diagnostic errors [1,7]. This highlights the importance of correlating pathological findings with clinical progression and maintaining a high index of suspicion in atypical presentations. In retrospect, the initial diagnosis of cholesteatoma can be explained by both the overlapping clinical features and the limitations of a non-representative biopsy, a phenomenon known as diagnostic anchoring. Notably, the highly vascular and friable appearance of the lesion, which is atypical for cholesteatoma, represented an important clinical clue that should have warranted further investigation. According to the World Health Organization Classification of Tumors and the ICD-O coding system, this tumor is classified as osteosarcoma, NOS (ICD-O code 9180/3) [8].

Reported cases of head and neck osteosarcoma, particularly those involving the temporal bone, are associated with variable outcomes depending on tumor grade, resectability, and the use of multimodal therapy. Surgical resection with negative margins remains the cornerstone of treatment when feasible, while adjuvant radiotherapy and chemotherapy have been associated with improved local control in high-grade tumors. Nevertheless, recurrence rates remain high, especially in anatomically complex regions such as the skull base, where achieving complete resection is often not feasible [3,4].

Compared to previously reported cases, our patient demonstrated early recurrence and rapid intracranial progression, reflecting the aggressive biological behavior of high-grade sarcomas. Nevertheless, the use of combined radiotherapy and systemic chemotherapy resulted in meaningful clinical benefit and tumor cytoreduction, consistent with outcomes described in the literature for patients managed with multimodal approaches.

The coexistence of pregnancy significantly complicated management. Treatment decisions required balancing maternal prognosis with fetal safety, necessitating individualized and multidisciplinary approaches. In this case, the initiation of radiotherapy during pregnancy, as referenced by the International Commission on Radiological Protection, followed by early delivery, reflects the prioritization of maternal oncologic control in the setting of rapidly progressive disease [9]. Ultimately, this case illustrates the complexity of real-world clinical practice, where diagnostic uncertainty, disease aggressiveness, and patient-specific factors converge, demanding flexible and multidisciplinary management strategies. Despite the constraints imposed by pregnancy, the timely initiation of radiotherapy and subsequent systemic treatment allowed for meaningful disease control without compromising maternal outcome.

A limitation of this case is the absence of molecular profiling and proliferation index assessment, which could have provided additional prognostic and therapeutic information.

## Conclusions

Temporal bone osteosarcoma is an exceptionally rare entity that may clinically mimic benign otologic conditions such as cholesteatoma. This case highlights the potential for diagnostic error when relying on limited biopsy samples and emphasizes the importance of integrating clinical, radiological, and pathological findings.

Furthermore, this case underscores the aggressive nature of high-grade sarcomas in the head and neck region and the challenges associated with achieving both a definitive diagnosis and appropriate management. The coexistence of pregnancy adds an additional layer of complexity, requiring multidisciplinary coordination and individualized therapeutic strategies. Early recognition and prompt intervention remain essential to improving outcomes in these rare but highly aggressive tumors.
